# A survey of college students’ willingness to participate in social practice with perceived environmental support based on the applied mixed research method

**DOI:** 10.3389/fpsyg.2022.972556

**Published:** 2022-09-08

**Authors:** Yingxin Li, Zhou Jin, Gaoqi Dong, Ran Zheng, Ting Wang

**Affiliations:** ^1^College of Educational Science and Technology, Zhejiang University of Technology, Hangzhou, China; ^2^College of Science and Technology, Ningbo University, Cixi, Zhejiang, China

**Keywords:** theory of planned behavior, mixed research method, social practice, participation willingness, perceived environmental support

## Abstract

Contemporary social reform promotes rapid social transformation, and social practice has a special educational function in higher education. However, research shows weak willingness to participate in social practice among college students. Using the mixed research method, 438 completed questionnaire surveys on perceived environmental support were collected from college students. The influence of perceived environmental support on Chinese college students’ willingness to participate in social practice was analyzed using partial least squares structural equation modeling, and an empirical test was conducted. The findings are as follows: (1) Perceived environmental support significantly impacts students’ participation attitude and perceived behavioral control. (2) Participation attitude and perceived behavioral control significantly influence participation intention, but behavioral norms have no significant influence. (3) Participation intention and perceived behavioral control significantly influence actual behavior. This study provides the theoretical basis of perceived environmental support for future research on social practice participation intention and offers some theoretical guidance for the implementation of social practice in China.

## Introduction

Presently, social reform is promoting rapid social transformation, and social practice activities have undergone drastic changes in terms of mode and communication (Zheng and Yang, 2006). In China, in 2017, the Outline of Curriculum Guidance for Comprehensive Practical Activities in Primary and Secondary Schools established that comprehensive practical activities are compulsory courses stipulated by national compulsory education and ordinary high school curriculum programs. In Singapore, civic education emphasizes the concept of the unity of knowledge and action and upholds the educational principle of integrating theory with practice (Armstrong and Tsokova, 2019). In the United States, the service learning model, which closely approximates social practice, is used to strengthen the effectiveness of school social practice (Koenis, 1993). In Japan, social education is defined as an organized off-campus educational activity that undertakes lifelong education for adults after school education (Iwatsuki, 2019). In the United Kingdom, citizenship education is summarized under three themes: community participation, political literacy, and moral responsibility (Jerome, 2012). In Canada, university teachers require students to write reflection reports and engage in self-reflection and self-education through practical activities (Gemmel and Clayton, 2009). In today’s era, social practice, as a kind of social activity conducive to college learning and personal development (Zhao et al., 2005), has become an important means to promote the socialization of college students in various countries and regions around the world (Barretti, 2004), and it is an important part of college students’ quality-oriented education.

The existent problems in social practice have seriously affected the development of the educational role of social practice (Yao and Shi, 2016). Paul (2017) has pointed out that social practice faces the problem of utilitarianism in the value orientation of participation. Once students achieve their own goals through the benefits and value obtained from participating in social practice, they tend to discontinue their participation in social practice and may even approach it perfunctorily (Shaw and Reyes, 1992; Yang, 2015). Borden et al. (2008) pointed out that some students have a bad attitude when they participate in social practice. Makhmudov (2020) noted the lack of innovation regarding the specific content and form of social practice; Zupic and Čater (2015) asserted that the organization and management of social practice are unscientific, and Van Der Wiele (1995) pointed out that when the content and form of social practice are not innovative or its upper-level organization and management are unscientific and not properly arranged, students’ interest in learning and enthusiasm for participation will be greatly reduced, ultimately lowering their willingness to participate. These conditions, in turn, further stimulate students’ rebellious psychology toward social practice (Xu et al., 2016), manifesting a mental and spiritual burden (Paas et al., 2016) and resulting in the inactivation of students’ willingness to participate in social practice. Therefore, we use the theoretical framework of the theory of planned behavior (TPB) to explain the influence on college students’ intention to participate in social practice from the perspective of perceived environmental support.

This study believes that environmental support, as an influencing factor to promote students’ social practice participation behavior, can better explain students’ social practice participation intention and perceived behavioral control (PBC). According to past studies, tacit knowledge and experience are embedded in specific social groups and social situations and are related to environmental support and environmental management in social practice (Bresnen et al., 2003). In contrast to traditional individualistic and rationalist behavioral approaches, social practice is collective-centered, and it helps individuals realize perceptions, interpretations, and actions in social environments (Hargreaves, 2011). Klem and Connell (2004) showed that teacher support in social practice is related to students’ participation and sense of achievement. Baird et al. (2007) found that organizational management and organizational culture are important factors affecting the success of social practice activities. Saye and Brush (1999) used information technology to support students’ participation in social issues in the learning environment, strengthen students’ practical experience, and enhance students’ participation behavior.

According to the existing research, environmental support is an important factor to promote participation in social practice behavior. Regarding previous research on social practice, domestic and foreign research is mostly limited to the content of social practice; that is, it mainly discusses the logical structure (Ma, 2003), the core composition, and the value embodiment of social practice (Wang, 2005; Ma, 2007), etc., while research on students’ willingness to participate in social practice is non-existent. Based on the TPB (Ajzen, 1991), Rhodes and Courneya (2005) found that attitude, subjective norms, and PBC were linearly correlated with students’ intentions and behaviors. Also based on the TPB, Yean et al. (2015) confirmed that attitude, subjective norms, and PBC can affect individuals’ subjective intentions. Vosgerau and Newen (2007) pointed out that thought can trigger action, and individual subjective will plays a key role in influencing individual behavior. Therefore, this study focused on the perspective of perceived environmental support (Hwang et al., 2010), screened out the indicators of influencing factors of perceived environmental support and willingness to participate, and demonstrated the related influencing factors through a questionnaire survey to explain the influence of different factors on college students’ intention to participate in social practice. In sum, this study answers the following questions:

1. Which methods should teachers and schools use to enhance students’ willingness to participate in social practice?
2. Does students’ willingness to participate in social practice differ because of different attitudes, subjective norms, and perceived behavioral control?
3. Does students’ perceived environmental support vary according to the degree of support they receive from teachers, organizational management, and environmental facilities?
4. Does students’ actual behavior differ due to individual variance in perceived behavioral control?

## Literature review

### Theory of planned behavior

The TPB is an important theory in social psychology to explain and predict individual behavior. The core idea of this theory is that the individual’s subjective will plays a key role in influencing their behavior. The TPB has been widely used to explain and predict all kinds of behaviors. Ajzen (1991), the theory’s author, presented it as comprising three parts. The first is personal attitude toward behavior, where “attitude” refers to the actor’s determination of whether a certain behavior is personally beneficial or disadvantageous. Secondly, subjective norms represent third parties’ normative expectations, that is, how the people who are important to the actor expects them to behave (Rueda Barrios et al., 2022), including but not limited to family members, teachers, friends, etc.; common views on certain behaviors also positively impact individual behavior intentions (Liu, 2017). Finally, PBC is understood as the perceived ease or difficulty of performing a behavior. The relevant research on the TPB is shown in Table 1.

**TABLE 1:** Research on the theory of planned behavior (TPB).

References	Research background	Independent variables	Dependent variable	Basic theory
Cheng, 2019	Online learning	Subjective norms, behavioral control, self-esteem, perceived usefulness, perceived ease of use, and attitude	Continuous use intention	Technology acceptance model (TAM) + TPB
Lihua, 2021	College students’ entrepreneurship	Entrepreneurial intentions, attitudes toward entrepreneurs, subjective norms, perceived behavioral control, personality traits, personal values, and habits	Sustainable entrepreneurial intent	Entrepreneurial event model (EEM) + TPB
Shukri et al., 2022	Traffic behavior	Attitudes, subjective norms, perceived behavioral control, ethics, past violations, history of traffic accidents, and psychological stress	Intention to avoid violating traffic regulations	TPB
Wang et al., 2021	Secondary vocational school students’ further education	Attitudes, subjective norms, perceived behavioral control, and gender	Willingness to pursue further education	TPB
Skoglund et al., 2020	Lecture attendance	Attitudes, subjective norms, and perceived behavioral control	Willingness to attend lectures	TPB
Haunberger and Hadjar, 2020	Students’ course selection	Attitudes, subjective norms, perceived behavioral control, and gender	Willingness to take social science courses	TPB + gender orientation theory
Summers, 2021	Elective courses	Background variables, behavioral beliefs, personal attitudes, subjective norms, and perceived behavioral control	Willingness to take science courses	TPB

Rueda Barrios et al. (2022) conducted an in-depth study on sustainable entrepreneurship intentions among college students in northeast Colombia based on the TPB. Shukri et al. (2022) used the TPB to explain why drivers violate traffic rules. Attitudes are divided into cognitive and emotional attitudes, subjective norms are divided into prohibitive and descriptive norms, and PBC is considered to be composed of perceived difficulty and self-efficacy. Additionally, the research team successfully explained the causes of traffic rule violations by combining the TPB with moral norms and psychological stressors. Therefore, regarding participation attitude, this study includes students’ cognitive attitude toward social practice (Chaiklin, 2011) as well as their emotional attitude (Malti and Noam, 2016), and regarding subjective norms, this study includes prohibitive norms proposed by schools and teachers for social practice (e.g., during the novel coronavirus pandemic, cross-province mobility and the offline gathering of practice team members are prohibited) and descriptive norms (e.g., encouraging students to engage in environmental governance, science popularization, social research, development planning, and other community activities).

Presently, although the TPB is mainly used in the field of health care, many scholars have applied it to education. For instance, Skoglund et al. (2020) used the TPB to evaluate the factors that influence PharmD students’ willingness to attend lectures. Those scholars pointed out that the three components that influence behavioral intentions are further explained by three beliefs: Behavioral beliefs explain attitude, normative beliefs explain subjective norms, and control beliefs explain PBC. Haunberger and Hadjar (2020) combined “past behavior” and “identity” with the TPB when studying male students’ low willingness to choose social science courses, especially considering the influence of gender on their intention to study the social sciences. Summers (2021) conducted a survey on “behavior, related attitudes and prejudice against science,” using the TPB and a mixed method design to explain the gap between reported intentions and middle school students’ actual enrollment in elective science courses.

Therefore, the TPB is adopted in this study as the main research framework, the primary reason being that the TPB has provided an appropriate theoretical basis for educators in previous studies, and it exerts an important impact on the formation of students’ participation behaviors. The second reason is that participation in social practice is a planned behavior. Without proper planning, social practice cannot be smoothly executed. However, the TPB can explain and predict individual behavior.

### Perceived environmental support

Perceived environmental support includes three main aspects: teacher behavior support (Kelm and McIntosh, 2012), organization and management support (King, 2017), and support from environmental facilities (Harel and Papert, 1990).

Firstly, regarding teacher behavior support, in recent years, it has gradually been established that certain teacher behavior is conducive to improving teaching design (Sergis and Sampson, 2017), aiding teaching and learning (McKenney and Mor, 2015), and enhancing students’ interest in learning as well as their learning efficiency (Wang et al., 2020). The sequencing pedagogical structure (SPS) framework indicates that teachers’ guiding role in the teaching process can be a means of implementing the student-centered teaching philosophy and can improve the structural teaching sequence (Jacobson et al., 2015). Suldo et al. (2009) applied mixed research methods and found that teacher support in social practice can improve adolescents’ subjective well-being and give full play to the supporting role of teacher behavior in social practice.

Secondly, regarding organizational management support, effective forms of teaching organization need to be adapted to suit teaching objectives and teaching content (Winslow, 1996). Massy et al. (1994) pointed out that fragmented communication, resource limitations, and inappropriate evaluation hinder the smooth progress of activities and the improvement of teachers’ teaching. Berman (1977) believes that the proper organization and management of activities is the fundamental factor that affects the “kernel of educational practice.” Nasurdin et al. (2008) found that perceived environmental support mediates the relationship between human resource management and organizational commitment as well as that between student-specific practice and school macro guidance in social practice. Organization and management support includes agenda setting, transportation arrangements, social practice management measures, etc., which are necessary to improve the robustness and security of the platform (Xia, 2020) in order to improve the learning effect on as well as the motivation and satisfaction of learners (Holenko Dlab and Hoic-Bozic, 2021).

Finally, regarding support from environmental facilities, it is important to note that social practice is part of the learning environment. With face-to-face or virtual access to learning materials, students can engage in learning activities and evaluate the learning process (Yousafzai et al., 2016). Research has proven that a good teaching environment can deepen students’ interest in learning, promote their engagement in cognitive and psychological activities during the learning process, and improve students’ behavioral intentions (Christie and De Graaff, 2017). Smith and Hardaker (2000) realized innovation in distance learning in the Internet-based learning environment, promoted students’ personalized development, and used the transferability of learning technologies and methods to support social practice. Chuang (2014) helped students adapt to the technology-supported learning environment by improving learning motivation and student participation. Social practice learning is special because the learning environment is not limited to school. Learners can learn anytime and anywhere through social practice (Hamidi and Chavoshi, 2018), and they can also obtain a variety of learning materials (Martin and Ertzberger, 2013) from the environment, while possibly cultivating various abilities and seeing improvement (Jiang, 2021).

Social practice, as a medium connecting schools and society, plays an important role in promoting students’ development (Cuiping, 2021). Presently, there are many objective evaluation methods to encourage college students to participate in social practice, but to get students more actively involved in social practice—-specifically, to encourage college students to participate in social practice activities linked to their study intentions—-it is also necessary to explore issues related to and collect and analyze students’ perceptions of environmental support for their achievements. In the literature, there are few TPB-based studies that explore college students’ intentions to participate in social practice activities. Therefore, to fill this theoretical gap, the concept of perceived environmental support is used in this study as a pre-factor affecting manifestation of the TPB.

## Research model and hypothesis

### Research model

**FIGURE 1:**
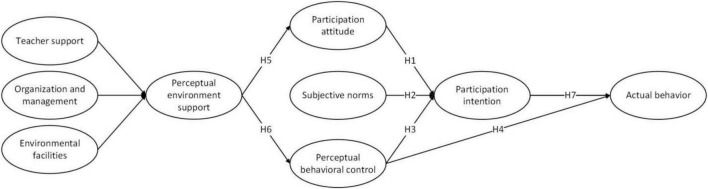
Research model.

In a research situation where college students are required to participate in social practice, the proposed research framework to understand the influence of the TPB on social practice participation intentions is diagrammatically represented in Figure 1.

### Research hypothesis

#### Participation intention

The main elements of the TPB are attitude toward participation, subjective norms, and PBC. According to the TPB, these three elements directly impact individuals’ participation intentions. When students have a positive attitude toward participation and the support of important people (Fielding et al., 2008) such as teachers, family members, partners, etc., the combination positively impacts their social practice participation, as well as students’ belief that social practice is valuable and easy to accomplish, which can produce positive PBC, all of which, in turn, may positively impact students’ participation intentions. Additionally, students with strong personal abilities have stronger PBC and are more likely to have positive participation intentions.

According to the TPB, the expected effect of personal attitude on behavior and the perceived effect of these expectations will affect the result of behavior (Duan and Jiang, 2008). A person’s attitude toward the occurrence of individual behavior and the corresponding results is called their behavioral attitude, which is mainly divided into two parts: One is belief intensity, and the other is evaluation of the results of the behavior (Zsóka et al., 2013). The connotation of the former refers to the degree of clarity regarding the behavior. The greater the belief intensity, the more positive the participation attitude. The latter refers to subjective estimation of the consequences and influences of the actions to be taken. The higher the individual’s expectation of success as a result of performing the behavior or the more severe the estimation of its consequences, the more positive or negative the individual’s attitude toward participation, respectively. These two components combined affect the individual’s participation attitude. Based on the above discussion, this study proposes Hypotheses 1–3:

H1: Participation attitude has a positive influence on participation intention.

H2: Subjective norms have a positive impact on participation intention.

H3: Perceived behavioral control has a positive impact on participation intention.

#### Environmental support

Luo et al. (2012) and others have asserted that learning attitude is students’ relatively stable psychological tendency to engage in learning and with their learning situations. Students’ attitudes are a key factor to cultivate more and better learning and improve their academic performance, while a good learning environment is crucial to maintaining students’ positive attitude (Ruiz-Jiménez et al., 2022). PBC reflects both internal and external factors (Kinnear et al., 1974) and depends on the resources and opportunities available at a given time. The higher an individual’s dependence on their environment, the lower their behavioral control (Ajzen and Madden, 1986). Based on the above discussion, this study proposes Hypotheses 5 and 6:

H5: Environmental support has a positive impact on participation attitude.

H6: Environmental support has a positive impact on perceived behavioral control.

#### Actual behavior

Dialectical materialism holds that practice is the foundation of cognition, and cognition negatively affects practice. Social practice has reformed the higher education teaching process regarding aspects of its influence on educational practice (Hashim and Ho, 2011). Some scholars have researched environmental behavior and awareness (Nechita et al., 2014). For instance, Uzun and Keles (2012) studied the relationship between environmental awareness and behavior, and Zsóka et al. (2013) studied Hungarian students’ environment, attitudes, and behavior. Conscious behavior is rooted in perception (Ni, 2021), and the expression of behavioral awareness needs to be controlled through perception. Based on the above discussion, this study proposes Hypotheses 4 and 7:

H4: Perceived behavioral control has a positive influence on actual behavior.

H7: Participation intention has a positive influence on actual behavior.

### Questionnaire design

This study drew upon the TPB to design a questionnaire, with reference to the extant literature (Ajzen, 1991; Godin and Kok, 1996; Conner and Armitage, 1998). The questionnaire contains nine questions concerning perceived environmental support. Scale design follows Jacobson et al. (2015) and other relevant literature. Perceived environmental support is composed of three main second-order sub-facets, with the following question distribution: three questions on teacher behavior support, three questions on organization and management support, and three questions on support from environmental facilities. Secondly, the TPB was applied according to Al-Jubari’s (2019) scale. The questionnaire includes three questions each on participation attitude, subjective norms, PCB, participation intention, and actual action.

Before questionnaire administration, this study conducted a pilot test to improve the questionnaire’s content validity by avoiding ambiguity and inappropriate questions. A total of 231 people participated in the pilot test. Cronbach’s α for internal consistency was used to perform reliability analysis of the questionnaire’s indicators. The results show that the Cronbach’s α values for all indicators exceeded 0.7. Regarding the pilot test, the Cronbach’s α values were between 0.757 and 0.956, and the standard values exceeded 0.7. Therefore, the questionnaire was deemed fit to be distributed.

## Data analysis

### Data collection

To verify the influence of perceived environmental support on students’ willingness to participate in social practice, this study administered a questionnaire survey to college and university students who participated in social practice activities and possessed a certain level of knowledge as well as logical discrimination ability in 2022. A total of 452 questionnaires were collected through the Questionnaire Star system (WJX). Following Jin et al. (2021), Lin et al. (2021), and Su et al. (2021), the questionnaire took at least 3 min to complete, and completed questionnaires were treated as invalid if responses to items were identical or in cases where extreme answers were provided. Fourteen invalid responses that were traced to duplicate login accounts and/or were completed in less than 30 s were deleted, resulting in 438 valid questionnaires, which were used for data analysis.

Regarding the respondents’ demographic characteristics, there were 233 male and 205 female respondents. Among these, 149 were liberal arts students, 134 were science students, and 155 were engineering students. Regarding level of study, 281 undergraduate and 157 graduate students participated in the questionnaire survey, accounting for 64.2 and 35.8% of the total number of respondents, respectively. Among them, the northern region sample numbered 123, accounting for 64.2% of the total number of questionnaires, while the southern region sample numbered 245, accounting for 54.20%; the northwest China sample numbered 47, accounting for 10.40%; and the Qinghai-Tibet area sample numbered 23, accounting for 5.09%. Statistical tabulation of respondents’ basic personal information is shown in Table 2.

**TABLE 2:** Summary of respondents’ demographic characteristics.

Attribute	Group	Number of people	Percentage of total sample
Gender	Male	233	53.20%
	Female	205	46.80%
Level of study	Freshman	64	14.61%
	Sophomore	71	16.21%
	Junior	78	17.81%
	Senior	68	15.53%
	Grade 1 master’s	61	13.93%
	Grade 2 master’s	63	14.38%
	Grade 3 master’s	18	4.11%
	Doctorate	15	3.42%
Area of study	Liberal arts	149	34.02%
	Science	134	30.59%
	Engineering	155	35.39%
Region	Northern China	123	27.21%
	Southern China	245	54.20%
	Northwest China	47	10.40%
	Qinghai-Xizang	23	5.09%

### Data analysis

This model examines the relationship between latent and explicit variables, and therefore, confirmatory factor analysis (CFA) was used to explore the questionnaire’s reliability. CFA is a mainstream analytical approach that includes factor loading, Cronbach’s alpha, composite reliability (CR), and average variance extracted (AVE). First, this study examined the factor loadings of the corresponding questionnaire items in SmartPLS. Normally, standardized factor loadings are higher, and the value must exceed 0.7 (Hair et al., 2017). The results of the factor loadings in the present study are shown in Table 3, with all factor loadings exceeding 0.7; therefore, each measured item exhibited good structural validity.

**TABLE 3:** Results of factor analysis.

Variable	Measurement	Factor loading	T
Teacher behavior support	TB1	0.894	48.402
	TB2	0.767	47.747
	TB3	0.764	38.71
Organization and management support	OM1	0.922	56.369
	OM2	0.891	53.366
	OM3	0.889	57.354
Support from environmental facilities	EF1	0.864	59.323
	EF2	0.815	59.35
	EF3	0.821	52.702
Perceived environmental support	PE1	0.823	59.244
	PE2	0.877	45.151
	PE3	0.853	46.225
Participation attitude	PA1	0.800	51.153
	PA2	0.841	38.349
	PA3	0.886	43.555
Subjective norms	SN1	0.829	41.589
	SN2	0.855	49.556
	SN3	0.860	50.506
Perceived behavioral control	PB1	0.823	57.867
	PB2	0.803	50.165
	PB3	0.837	58.358
Participation intention	PI1	0.850	59.013
	PI2	0.855	56.4
	PI3	0.812	52.878
Actual behavior	AA1	0.851	49.751
	AA2	0.878	64.521
	AA3	0.884	52.599

Hair et al. (2006) proposed that the reliability of internal consistency should be evaluated according to two indicators, namely composite reliability and Cronbach’s alpha, both of which should exceed 0.7. Table 4 shows that all the comprehensive reliability values exceeded 0.70, indicating good internal consistency. Combined reliability (CR) refers to the reliability of a combined variable (a new variable comprising the sum of more than one variable), and the value is required to surpass 0.5 (Charter, 2003). Fornell and Larcker (1981) proposed that AVE is an indicator of the dispersion between statistical sampling value and expected value. According to the recommendations in the literature, AVE should exceed 0.5. Table 4 shows that in the present study, all AVE values exceeded 0.5, indicating perfect convergence validity.

**TABLE 4:** Reliability and validity analysis results – CR, Cronbach’s alpha, and AVE.

Variable	Cronbach’s alpha	rho_A	CR	AVE
Subjective norms	0.806	0.809	0.885	0.719
Participation attitude	0.795	0.8	0.88	0.711
Participation intention	0.79	0.793	0.877	0.704
Actual behavior	0.841	0.841	0.904	0.759
Perceived environmental support	0.809	0.812	0.887	0.724
Teacher support	0.737	0.755	0.851	0.657
Environmental support	0.782	0.788	0.872	0.695
Perceived behavioral control	0.759	0.759	0.861	0.674
Organization and management support	0.884	0.885	0.928	0.811

The purpose of discriminant validity is to test the degree of discrimination between different constructs. Fornell and Larcker (1981) proposed that the square root of the AVE between different constructs should be greater than their correlation coefficient. In the correlation coefficient matrix for each construct, shown in Table 5, the diagonal is the square root of the AVE. As shown in Table 5, the square roots of the AVE values are all greater than the correlation coefficients between the constructs, indicating that the results for each construct have discriminant validity.

**TABLE 5:** Discriminant validity (Fornell and Larcker, 1981).

	Subjective norms	Participation attitude	Participation intention	Actual behavior	Perceived environmental support	Teacher support	Environmental support	Perceived behavioral control	Organization and management support
Subjective norms	0.848								
Participation attitude	0.610	0.843							
Participation intention	0.556	0.685	0.839						
Actual behavior	0.614	0.616	0.744	0.871					
Perceived environmental support	0.698	0.759	0.632	0.610	0.871				
Teacher support	0.515	0.518	0.509	0.542	0.549	0.811			
Environmental support	0.461	0.540	0.467	0.589	0.409	0.521	0.834		
Perceived behavioral control	0.576	0.592	0.632	0.639	0.561	0.598	0.661	0.821	
Organization and management support	0.378	0.466	0.498	0.577	0.469	0.791	0.706	0.645	0.901

Hair et al. (2017) proposed the heterotrait-monotrait (HTMT) correlation ratio to evaluate discriminant validity. Often used to assess discriminant validity, HTMT is a value generated by comparing the mean of each construct (based on uniform load) with its square uniform construct correlation. If the HTMT value is less than 0.90, discriminant validity exists between the two constructs. The maximum HTMT value in this study was 0.890. HTMT results showed that the student sample met all criteria. As shown in Table 6, this model has good reliability and validity.

**TABLE 6:** Differential validity (HTMT).

	Subjective norms	Participation attitude	Participation intention	Actual behavior	Perceived environmental support	Teacher support	Environmental support	Perceived behavioral control	Organization and management support
Subjective norms									
Participation attitude	0.766								
Participation intention	0.693	0.867							
Actual behavior	0.745	0.754	0.811						
Perceived environmental support	0.861	0.843	0.794	0.742					
Teacher support	0.673	0.680	0.669	0.696	0.705				
Environmental support	0.577	0.678	0.581	0.713	0.506	0.710			
Perceived behavioral control	0.736	0.758	0.810	0.797	0.713	0.810	0.862		
Organization and management support	0.460	0.555	0.592	0.669	0.555	0.890	0.858	0.787	

### Structural model

**FIGURE 2:**
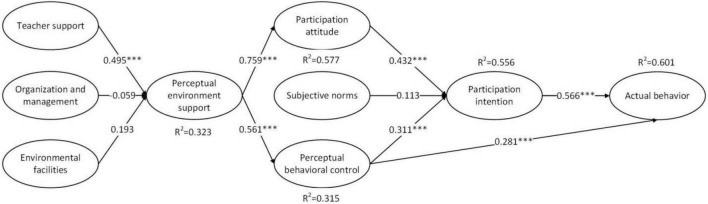
Structural model. ***At the 0.01 level (double tail), the correlation is significant.

This study adopted the bootstrapping re-sampling method with 5,000 samples to evaluate partial least squares (PLS) regression results (Hair et al., 2017). The results of the structural model are shown in Figure 2. Regarding individual constructs’ explanatory power, the *R*^2^ values for participation attitude, PCB, participation intention, actual behavior, and perceived environmental support were 57.7, 31.5, 55.6, 60.1, and 32.3%, respectively. This study can therefore be said to have a model with good explanatory power. In previous studies, under the TPB, the variance used to explain the influence of behavior was only 40–50%, and the explanatory power of the variance in behavior was even lower at only 19–38% (Sutton, 1998). Therefore, the present study’s framework has better explanatory power for intention to participate in social practice.

Regarding the results of verification of H1–H8, the statistical results for H1 and H4–H7 show support for these hypotheses. Among them, in the TPB, participation attitude (H1) and PCB (H3) significantly influence social practice participation intentions. Notably, subjective norms (H2) have no significant impact on entrepreneurial intention. Secondly, a significant relationship was found between perceived environmental support and participation attitude (H5) and PCB (H6); specifically, the coefficient for the influence of perceived environmental support on participation attitude was high (β = 0.759), but that of PCB was low (β = 0.561). Additionally, teacher support (β = 0.495) had a higher influence coefficient on perceived environmental support than organization and management support (β = 0.059) and support from environmental facilities (β = 0.193). Finally, both PCB (H4) and participation intention (H7) were found to significantly affect the actual behavior of participating in social practice.

## Discussion and conclusion

### Research discussion

The results of this study indicate that teacher support significantly impacts perceived environmental support, while organization and management support and that from environmental facilities exert no significant impact on perceived environmental support, and whether teachers effectively participate in social practice indirectly impacts students’ intention to participate (Girard et al., 2021). Support from the surrounding environment that students can easily perceive in social practice comes from the teachers who guide social practice (Affuso et al., 2022); therefore, instructors’ initiative should be given full play in social practice (Lynch et al., 2013). When the instructors involved in social practice select a research direction that is suitable for their students, students’ intention to participate in social practice will improve.

This study also found that perceived environmental support significantly impacts participation attitude and PCB. When students perceive environmental support for their participation in social practice, they are more likely to have a positive attitude toward social practice participation (Goldner and Golan, 2017) and be able to maintain their subjective attitude. However, it is worth noting that the influence of perceived environmental support on participation attitude is significantly stronger than that of PBC, which indicates that once students perceive external support, it is easier to maintain rather than change their original participation attitude.

The results also show that attitude and PBC significantly affect participation intention. The reason for this may be that students are more willing to participate in social practice when it reflects their interests (Daily, 2018) or when their behavioral beliefs are compatible with it. Therefore, to increase students’ willingness to participate in social practice, social practice content should be carefully selected, and students should be encouraged to communicate their beliefs and interests. A positive participation attitude may follow participation intentions, and students’ PBC can help maintain their participation intentions. On the contrary, students’ subjective norms cannot fundamentally change their intentions; subjective norms can only contribute to the habitual maintenance of students’ attitudes toward social practice (Van De Mieroop et al., 2021). If students’ attitudes toward social practice are negative, subjective norms will further deepen their ideological consciousness, nullifying their participation intentions.

Finally, PBC and participation intention significantly influence students’ practical action, suggesting that after social practice participation, students will be more willing to actually perform the action, will be capable of exercising self-control to avoid violating their personal behavioral code (Kammigan, 2022), and will be able to maintain a certain level of understanding. The final stage of social practice should therefore give students full play regarding their participation, help students solidify their own thinking and understanding rather than encouraging a negative attitude toward social practice work, and through increased PBC, guide students to improve their own actual participation in social practice activities and work toward meeting their developmental needs.

In view of the above conclusions, we offer the following suggestions: (1) In terms of external support, instructors’ initiative should be given full play in social practice. (2) During the early stages, guide students to establish an appropriate attitude toward participating in social practice, encourage students to actively participate in social practice, and expose students such that they witness the knowledge and skills that social practice participation yields. (3) Regarding the content of activities, encourage students to communicate their beliefs and interests. (4) Regarding final participation, students’ willingness to participate should be given full play. Students should also be encouraged to adhere to their own ideas and understanding and guided to participate in social practice activities for their own improvement and to meet their developmental needs through improved PBC.

### Practical and theoretical implications

#### Practical implications

Firstly, this study has referential significance for schools and educators regarding guiding students’ social practice. Through in-depth analysis of the theoretical model, this study answers the question “What influences students’ social practice participation intentions and actions?” (Wong and Hodson, 2010), which is pertinent because it represents a problem that higher education professionals are facing today. Relevant to present times, Tuomela (2002) recently explained social practice from the perspective of the mental state of social interweaving; Zheng and Wang (2010) observed that relevant workers in higher education are thoughtfully reflecting on strategies to improve the value of social practice; and Mukherjee et al. (1998) emphasized promoting improved social practice quality through knowledge driving and purposeful efforts to improve personnel training quality and enhance the level of scientific research as well as the social service ability. Based on the results of this study, higher education professionals can target challenges in social practice, innovate the forms that college students’ social practice participation takes, enrich the connotations of social practice, and increase its value (Li and Hao, 2020).

Secondly, this study investigated students’ satisfaction with social practice, which can provide guidance for solving problems such as “students’ insufficient awareness level” (Kuang et al., 2017) and their “negative mentality” (Yang, 2005) in their approach to social practice. Kiesewetter et al. (2016) pointed out that social practice is a better solution when students lack the knowledge to solve practical problems. Biggs (1993) adopted the cognitive system method to deduce teaching and learning theory from practice and carry out feasible teaching practice. Hayes and Allinson (1998) pointed out that practice as an effective intervention can improve individuals’ and organizations’ learning performance. MacBeth et al. (2008) used social mindset theory (Hermanto and Zuroff, 2016) to verify that stronger and more numerous interpersonal relations are associated with higher levels of paranoia, attachment, and avoidance; Yuan-Qiang et al. (2019) asserted that positive social mentality can clarify the goal, mechanism, and mode of social practice; and Harr (2013) pointed out that reducing the negative impact of excessive compassion and improving compassion satisfaction can better equip students to face social practice. Through teachers’ guidance, students can adjust their attitude and become more active, while deepening their understanding of the importance of social practice, so as to gain more from participation.

The purpose of studying the factors influencing social practice intentions and actions is to help college students “integrate into society” (Skop et al., 2021). Social practice is not only an important part of college students’ social integration but also an important component of tertiary education. Brandt and Clinton (2002) advise that social practice should avoid geographical limitations and that its advantages should be given full play in the local and regional context to help students better integrate into society. McMahon and Bhamra (2012) believe that students’ social practice should be sustainably integrated into their socialization training, so as to form a mechanism combining science and practice. Thyer (2015) has encouraged students to integrate theoretical research into practical practice, as it eases employment in terms of convenience and helps students more quickly adapt to society after their studies. For both higher education professionals and students, this study aims to enhance social practice. College students’ participation in social practice is an important means of directly promoting their transition from school to society. Moreover, college students’ participation in social practice can deepen their understanding of industry, society, and nation.

#### Theoretical implications

This study experimentally expands and combines the TPB and the theory of perceived environmental support. The traditional TPB claims only that participation attitude and perceived behavioral norms will affect individuals’ behavioral intentions (Duan and Jiang, 2008); however, the influencing factors of participation attitude and perceived behavioral norms have not been thoroughly studied. In this study, an environmental support variable was incorporated into the theoretical framework in combination with the TPB to compensate for the deficiency.

Second, this study supplements the TPB in the field of education. Presently, the TPB’s main application is in health care (Kan and Zhang, 2015), and there are few studies linking it to education. It is important to study and offer guidance on students’ intentions to learn as well as their practice behaviors, and the TPB can be useful in such research (Sun, 2021). As a pre-factor of the TPB, perceived environmental support can better explain students’ intentions to participate in behavior in the field of education.

### Limitations and future research

This study has some limitations and unresolved problems.

First, the impact of organization and management support and support from environmental facilities needs to be further clarified. This study concluded that perceived environmental support significantly impacts participation attitude and PBC, which indicates that it is beneficial to study the impact of environmental support on participation attitude. Humans’ social environment includes the physical environment, social relations, and the cultural environment (Casper, 2001), and the interaction between social context and individual characteristics in the social practice development process should be fully considered (Jordan, 2000). However, among the influencing factors of environmental support, only teachers’ support plays a significant role, while organization and management support and support from environmental facilities do not have a direct and significant role. The dependent variables affecting perceived environmental support need to be studied further.

Second, it is necessary to explore the specific causes of the formulation of behavioral beliefs. Multiple factors affect behavior and belief (Shahi et al., 2020), including which are belief and intensity and the consequences of behavior (Ajzen et al., 2004). However, studies have pointed out that behavioral beliefs play an important role in influencing behavior and participation intention in social practice (Gagné and Godin, 2000), subjective beliefs are crucial to behavioral reasoning (Sahu et al., 2020), and behavioral beliefs not only stimulate theoretical innovation but also provide behavioral meaning on the basis of facts (Danz et al., 2022). Therefore, this aspect also has high research value.

Third, personal ability needs to be further refined. Ability is a multidimensional concept, and achievement is closely related to ability and motivation (Dweck, 2002). Different learning situations can produce different kinds of specific abilities, leading to the differentiation of abilities (Ferguson, 1954). Therefore, it is necessary to further define abilities and refine the influence of different aspects of abilities on participation intention.

Fourth, guidance in the context of social practice should not raise unrealistic expectations but rather focus on creating opportunities to exercise social practice and reasonably arranging the associated process (Roberts, 1977). This study aims to propose a new model to study the influencing factors of students’ social practice behavior and participation intentions. Preliminary data have been collected on various factors’ degree of influence, thus enhancing the reliability of the theoretical basis for a follow-up study on specific practice activities.

## Data availability statement

The original contributions presented in this study are included in the article/supplementary material, further inquiries can be directed to the corresponding author.

## Author contributions

YL: formal analysis, investigation, and writing – original draft and review and editing. ZJ: methodology, investigation, visualization, and writing – review and editing. GD: conceptualization and writing – original draft. RZ and TW: writing – original draft and review and editing. All authors contributed to the article and approved the submitted manuscript.
